# Comparison of three contrast agents in the diagnosis of cracked teeth using Cone Beam Computed Tomography (CBCT)

**DOI:** 10.1038/s41598-026-54779-4

**Published:** 2026-05-24

**Authors:** Mawadah K. Alnaseri, Ruaa A. Alamoudi, Hanadi Sabban, Abdullah A. Albassam, Salem Alamri

**Affiliations:** 1 Department of Endodontics, Faculty of Dentistry, King Abdulaziz University, Jeddah, Saudi Arabia; 2 Department of Oral Diagnostic Sciences, Oral Radiology Division, Faculty of Dentistry, King Abdulaziz University, Jeddah, Saudi Arabia; 3 Department of Radiology, King Abdulaziz University Dental Hospital, King Abdulaziz University, Jeddah, Saudi Arabia

**Keywords:** Cracked teeth, CBCT, Contrast-enhanced CBCT, Contrast agents, micro-CT, Diseases, Health care, Medical research

## Abstract

Cracked teeth are difficult to detect on conventional cone beam computed tomography (CBCT). Contrast agents may improve visualization of fine and superficial cracks. This study compared three contrast agents for their diagnostic performance. Thirty-nine extracted premolars with induced micro-cracks were examined using three contrast agents: ioversol, barium sulfate, and meglumine ioxitalamate. Each tooth underwent CBCT before and after contrast application, followed by micro-computed tomography (micro-CT) as the reference standard. Two blinded observers recorded the number of cracks detected on CBCT and confirmed by micro-CT. Statistical analysis was performed using ANOVA with LSD post hoc comparisons. Micro-CT detected an average of 19.9 ± 9.3 cracks per tooth. On CBCT, ioversol and barium sulfate performed similarly (5.6 ± 2.3 and 5.8 ± 2.1 cracks, respectively), whereas meglumine ioxitalamate detected fewer (3.4 ± 1.7, *p* = 0.001). When matched against micro-CT, ioversol and barium sulfate detected ~ 4 cracks, while meglumine ioxitalamate detected fewer than 3. Barium sulfate detected the largest proportion of deep cracks (20.7%). Contrast-enhanced CBCT improves detection of fine cracks compared with unenhanced scans. Ioversol and barium sulfate demonstrated superior diagnostic value, while meglumine ioxitalamate offered limited benefit.

## Introduction

Cracked tooth syndrome is a common diagnostic challenge in clinical practice. Cracks may arise from structural weakness in the tooth, excessive occlusal loading, or as a consequence of restorative procedures^1–3^. Their prevalence tends to increase with advancing age and with a history of restorative treatment, and identifying these defects is critical for both treatment planning and long-term tooth preservation^4–6^. Failure to detect early cracks may allow progression to pulpal or periapical disease, ultimately resulting in tooth loss^7,8^.

Several traditional methods have been employed in the detection of tooth cracks, including transillumination, the use of dyes, bite tests, and magnification. Although these approaches are accessible in daily practice, their diagnostic value is inconsistent. Subtle or superficial cracks are particularly difficult to detect, and both sensitivity and reproducibility remain limited when these techniques are applied^9–11^. Conventional radiographs are likewise restricted; their two-dimensional nature and relatively low resolution prevent the direct visualization of fine structural defects, often leaving only indirect signs of damage^12,13^.

Recently, cone-beam computed tomography (CBCT) has been increasingly integrated into endodontic diagnostics, offering three-dimensional assessment of root and canal morphology^14–16^. Despite its advantages, CBCT has clear limitations in identifying narrow cracks. Image quality can be affected by factors such as voxel size, noise, and beam-hardening artifacts, all of which reduce the reliability of crack detection. As a result, reported diagnostic accuracy in this context is only moderate^17–19^.

Micro-computed tomography (micro-CT), by contrast, offers high-resolution imaging and has been adopted as the gold standard in experimental settings. Its use, however, remains confined to ex vivo studies because of technical and radiation considerations^20,21^.

The limitation of the sensitivity of CBCT in detecting fine structural defects has prompted growing interest in the application of contrast agents to enhance crack visualization.^22–24^. A recent systematic review and meta-analysis by Sabban and Abdel-Wahed (2025) confirmed that contrast-enhanced CBCT significantly improves diagnostic accuracy for detecting tooth cracks and fractures, highlighting its potential as a valuable adjunct in clinical practice^25^. Different contrast materials—including iodine-based compounds, barium-containing formulations, and organic agents—have been investigated, with evidence indicating that their use improves the delineation of subtle defects in extracted teeth^26–28^. The diagnostic efficacy of these contrast agents is largely determined by their physicochemical characteristics, such as particle size, viscosity, and osmolality^29,30^. Consequently, contrast-enhanced CBCT has been proposed as a supportive approach to clinical diagnosis of cracked teeth, although direct comparative data across different agents remain limited^31–33^.

The management of cracked teeth continues to present considerable clinical challenges and requires careful case-specific assessment. Superficial cracks confined to enamel can generally be managed conservatively, whereas fractures that extend into dentin or involve deeper tooth structures often require restorative or endodontic treatment. Prognosis is strongly influenced by both the accuracy and the timing of diagnosis^34–36^. Enhancing diagnostic precision through advanced imaging modalities may therefore improve clinical decision-making, facilitate tooth preservation, and reduce the risk of unnecessary extractions.

Although interest in contrast-enhanced CBCT has grown, few investigations have systematically compared different contrast agents, and the relative effectiveness of commonly available materials remains uncertain. To address this gap, the present study was designed to evaluate whether contrast enhancement improves the detection of fine and superficial cracks in extracted human premolars, using micro-CT as the reference standard. Three contrast agents—ioversol, barium sulfate, and meglumine ioxitalamate—were assessed to determine their comparative diagnostic performance.

Despite the increasing clinical application of CBCT, evidence directly comparing enhanced and unenhanced imaging for crack detection is still limited. Moreover, the diagnostic contribution of individual contrast agents has not been fully established.

Accordingly, the aim of this study was to compare the diagnostic performance of contrast-enhanced CBCT with conventional CBCT in detecting fine dentinal cracks, using micro-CT as the gold standard. The specific objectives were: (1) to evaluate and compare the diagnostic accuracy of three contrast agents—ioversol, barium sulfate, and meglumine ioxitalamate—for identifying narrow and superficial cracks, and (2) to determine the overall diagnostic gain of contrast-enhanced CBCT relative to unenhanced imaging.

Two null hypotheses were tested: (1) contrast-enhanced CBCT does not differ from unenhanced CBCT in its ability to detect cracks, and (2) there is no difference in diagnostic performance among the three contrast agents.

## Methods

### Sample size calculation

Sample size was calculated using STATA software (StataCorp, College Station, TX, USA), based on effect sizes reported by Hu et al^37^. An a priori power analysis with α = 0.05 indicated that a minimum of 35 teeth was required.

### Sample description

This in vitro study included 40 extracted human premolars collected from patients treated for orthodontic or periodontal purposes at the Faculty of Dentistry, King Abdulaziz University, Jeddah, Saudi Arabia. Ethical approval was obtained from the institutional review board by the Research Ethics Committee of the Faculty of Dentistry (RECFD), King Abdulaziz University, Jeddah, Saudi Arabia (Approval No. 124-06-23). All procedures were conducted in accordance with relevant guidelines and regulations, and written informed consent for the use of extracted teeth in research was obtained from all patients and/or their legal guardians.

Teeth were selected according to strict inclusion and exclusion criteria. Inclusion criteria comprised teeth without visible enamel cracks, root resorption, or severe abrasion. Exclusion criteria comprised teeth with carious lesions, vertical root fractures, wedge-shaped cervical defects, or a history of endodontic therapy or coronal restorations.

### Tooth preparation and crack simulation

All specimens were cleaned of residual soft tissue and calculus, then fixed in silicone putty to provide stability during procedures. Artificial cracks were created by thermal cycling: each tooth was immersed in liquid nitrogen (–196 °C) for 1 min, and subsequently in boiling water (100 °C) for 1 min. The process was repeated up to three cycles or until a visible crack developed. This method was adapted from previously validated protocols that established thermal cycling as a reliable approach for generating enamel–dentin cracks^38,39^. One specimen fractured during the crack simulation procedure and was therefore excluded from the study, resulting in a final sample of 39 teeth. All remaining specimens were then examined under magnification to verify the formation of cracks. Although crack morphology naturally varies between teeth due to factors such as enamel thickness or inherent structural defects, we standardized the process by applying identical thermal cycling parameters across all specimens. Each contrast agent was evaluated on the same post-cycling tooth.

### Micro-CT reference standard

All specimens were examined with micro-computed tomography (micro-CT), which served as the reference standard for crack detection^20^. Imaging was performed using a Bruker SkyScan 1172 high-resolution scanner (Bruker SkyScan, Kontich, Belgium) under standardized conditions: 92 kV, 72 µA, 474 ms exposure time, 17 μm voxel size, 0.4° rotation step, and frame averaging of four. To reduce beam hardening, Cu + Al filters were applied, and flat-field correction was included in the protocol. Reconstruction was conducted with N-Recon software (v1.6.9.4) applying a ring artifact reduction factor of 5, beam-hardening correction at 25%, and Gaussian smoothing set at 2. The reconstructed datasets were exported in 16-bit TIF format for subsequent evaluation. The reconstructed micro-CT images were evaluated to identify and document cracks within each specimen. Cracks were defined as linear discontinuities in the enamel or dentin visible across consecutive slices. Crack depth was determined using the micro-CT images. Cracks extending beyond the enamel–dentin junction and into the dentinal structure were classified as deep cracks. Each dataset was inspected with Dataviewer software (v1.5.6.2, Bruker SkyScan) to document the number, location, and extent of cracks.

### Application of contrast agents

To approximate intraoral conditions, specimens were immersed in artificial saliva (Chemazone BZ109) for 24 h prior to testing. Three contrast agents— ioversol (Liebel-Flasheim Company LLC, USA), meglumine ioxitalamate (Telebrix^®^, Guerbet, France), and barium sulfate suspension (GEHealthcare, Ireland)—were evaluated in sequence. The iodinated contrast agents were used according to their commercially available concentrations, while the barium sulfate was prepared as a homogeneous suspension prior to application.

Each agent was applied using a micro-brush in three successive coats covering the entire crown surface, with approximately one-minute drying intervals between applications to allow adequate surface penetration. Although exact layer thickness could not be quantitatively standardized, the same application technique and brushing pressure were used for all specimens to maintain consistency. A brushing protocol was selected based on previously reported experimental studies evaluating contrast-enhanced,^40^ where surface application allows contrast agents to penetrate crack interfaces without introducing excessive pressure or artificial infiltration.

To maintain blinding, the original labels were removed, and the agents were coded as A, B, and C, where material A corresponded to ioversol, B to meglumine ioxitalamate, and C to barium sulfate. After each application, CBCT imaging was performed between testing cycles. The specimens were then rinsed thoroughly and stored in saline overnight to eliminate any residual contrast agent that might interfere with subsequent tests. The duration of this storage period was determined based on a preliminary pilot experiment conducted prior to the main study, which indicated that overnight immersion was sufficient to allow complete removal of residual contrast material.

### CBCT imaging protocol

Baseline CBCT scans (unenhanced) were obtained using a KaVo 3D eXam CBCT unit (KaVo Dental GmbH, Biberach, Germany), operated at 90 kV, 6.3 mA, and 8.7 s, with a dose-area product of 509 mGy·cm². A limited field of view (5 × 5 cm) was applied, generating isotropic voxels of 0.125 mm. All image datasets were subsequently reviewed using OnDemand3D™ software (Cybermed Inc., Seoul, South Korea).

### CBCT examination

Two examiners, a board-certified oral and maxillofacial radiologist (HS) and a postgraduate endodontic student (MA)—independently evaluated axial, sagittal, and coronal CBCT reconstructions for the presence of cracks. Prior to data collection, calibration sessions were conducted to standardize interpretation criteria. Each examiner repeated the assessment one week later to assess intra-examiner agreement.

A crack was defined as a linear hypodense discontinuity in enamel or dentin visible on at least two consecutive slices and consistent across multiple reconstruction planes. When the same crack was visible on consecutive slices or different planes, it was recorded as a single crack based on its anatomical location and trajectory within the tooth structure. This definition is consistent with previously published diagnostic criteria^41,42^. Each identified crack was assigned a unique code and documented to ensure consistent tracking and to avoid double counting during comparison between CBCT and micro-CT datasets. Image processing tools, including filtering, brightness/contrast adjustment, and zoom, were applied when necessary to optimize visualization. Each detected crack was coded according to a standardized system (e.g., 30 A #3), and representative screenshots were archived for documentation.

To minimize false-positive results, strict exclusion criteria were applied:

- Location: linear features associated with pits, fissures, or the cementoenamel junction were disregarded.
- Density: poorly defined low-density lines resembling natural enamel–dentin interfaces were excluded.
- Size: features too small to be distinguished from imaging artifacts were not considered.

**Fig. 1:**
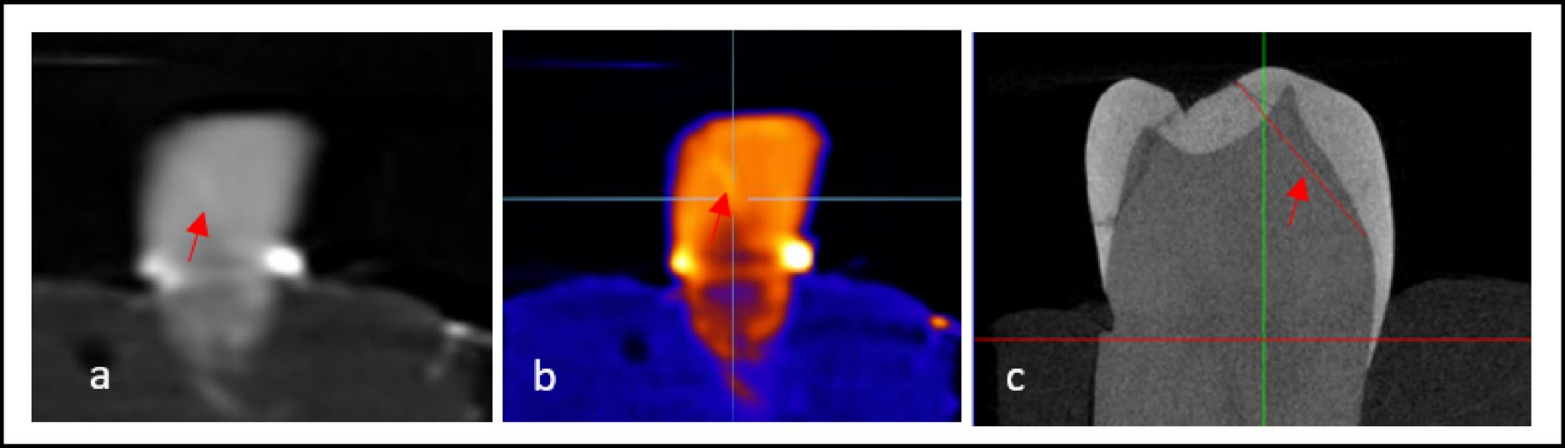
Comparison between unenhanced-CBCT image (**a**), and enhanced-CBCT image (**b**), and micro-CT (**c**) of representative tooth cracks. Arrows indicate the location and orientation of the detected crack.

**Fig. 2:**
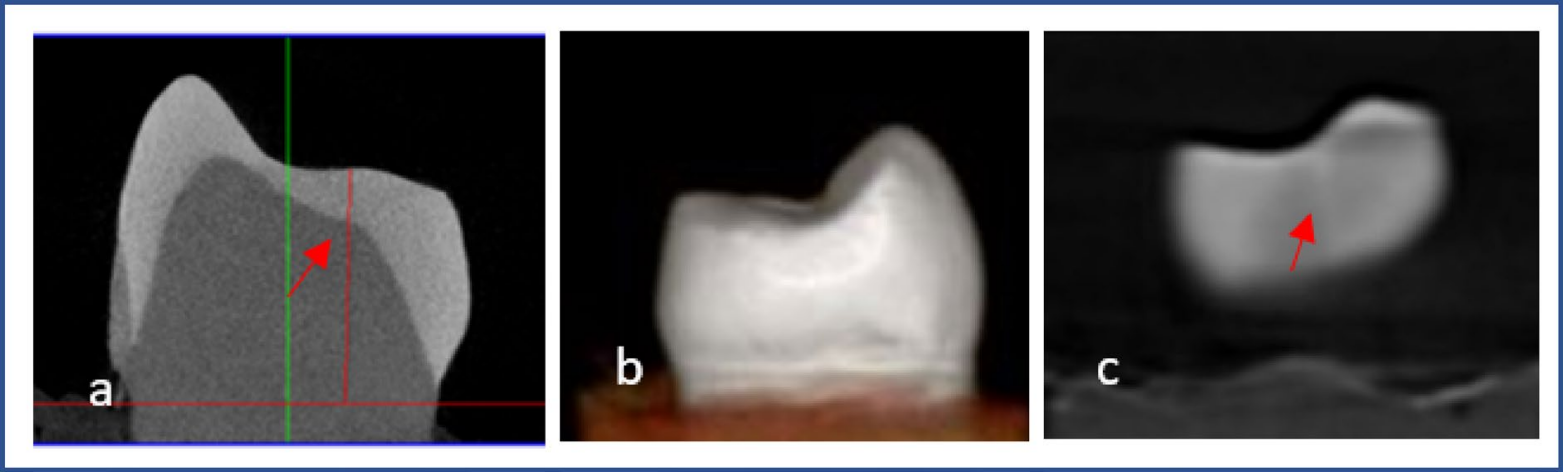
Comparison between micro-CT (**a**), unenhanced-CBCT (**b**) image, and enhanced-CBCT (**c**) of tooth cracks with barium sulfate enhancement. Arrows point to the crack lines, showing areas of improved visibility.

**Fig. 3:**
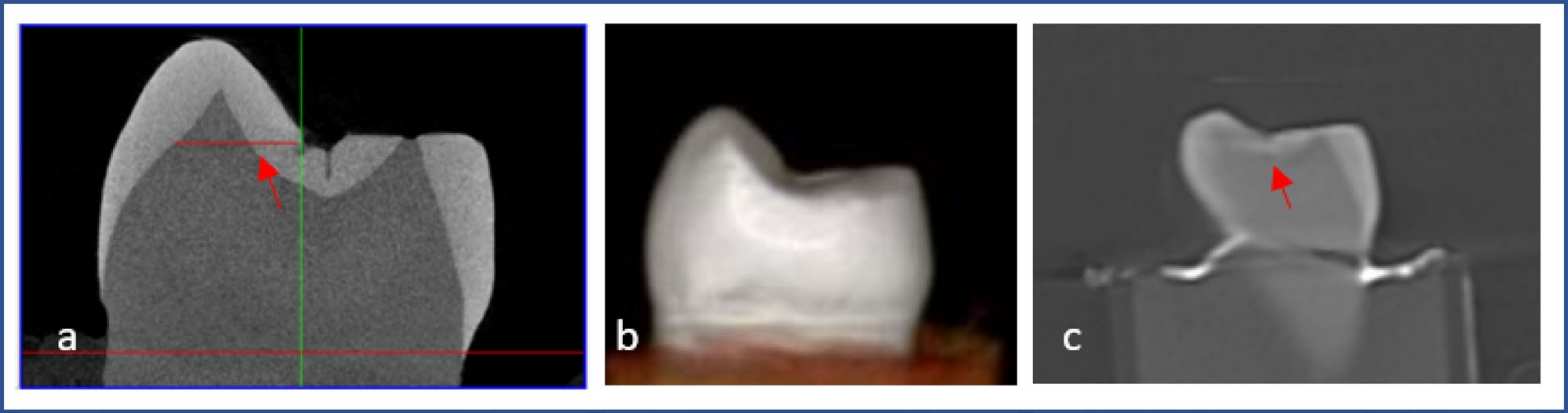
Comparison between micro-CT (**a**), unenhanced-CBCT image (**b**), and enhanced-CBCT image (c) of tooth cracks with ioversol enhancement. The direction of the arrow highlights the detected crack.

**Fig. 4:**
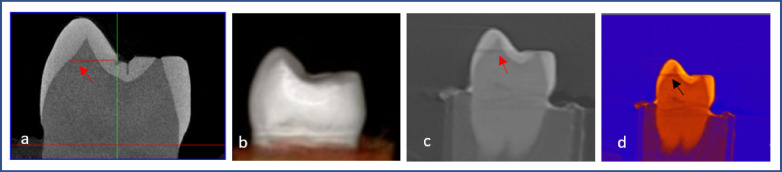
Comparison between micro-CT (**a**), unenhanced-CBCT (**b**), enhanced-CBCT image (**c**), and warm-filtered enhanced-CBCT image of tooth cracks with meglumine ioxitalamate enhancement. Arrows identify the direction of the crack.

These criteria were applied to prevent misinterpretation of normal anatomical structures or imaging artifacts as cracks and were used consistently across all imaging conditions to ensure standardized evaluation. Representative examples of cracks observed using micro-CT, unenhanced CBCT, and contrast-enhanced CBCT are shown in Figs. 1, 2, 3 and 4.

Discrepancies between the two examiners were resolved through consensus review. Following this process, one examiner (MA) aligned CBCT findings with micro-CT results. This matching step was conducted only after completion of the blinded CBCT evaluation to avoid influencing the initial crack detection. Matching was performed according to predefined criteria, in which a CBCT-detected crack was considered corresponding to a micro-CT crack when both the anatomical location and orientation of the crack were consistent after alignment of the datasets. Due to orientation differences between modalities, µ-CT slices were realigned to CBCT data using a specialized matching tool (application Rectangle v0.67, Ryan Hanson, 2019–2023), allowing for an accurate comparison of each detected crack (Figs. 1, 2, 3 and 4).

### Statistical analysis

All statistical procedures were performed using SPSS software, version 23.0 (IBM Corp., Chicago, IL, USA). Descriptive statistics were expressed as means with standard deviations for continuous variables and as frequencies with percentages for categorical data. Because the same specimens were evaluated under multiple conditions, repeated-measures ANOVA were applied to assess differences among groups. Pairwise comparisons were performed using the least significant difference (LSD) post hoc test. Inter- and intra-examiner agreement was quantified using intraclass correlation coefficients (ICC). Intraclass correlation coefficients were calculated using a two-way random effects model with absolute agreement (ICC(2,1)). 95% confidence intervals were calculated to assess the precision of the reliability estimates. A p-value ≤ 0.05 was considered to indicate statistical significance.

Because multiple cracks could occur within a single specimen, the outcome measure was defined as the number of cracks detected and confirmed against the micro-CT reference standard, rather than a binary classification of crack presence or absence. Therefore, comparisons between imaging conditions were performed based on crack counts.

## Results

###  Descriptive analysis

The mean number of cracks identified with micro-CT was (19.92 ± 9.26; 95% CI: 16.93–22.91). In comparison, CBCT with contrast enhancement detected markedly fewer cracks: ioversol (5.64 ± 2.32; 95% CI: 4.89–6.39), meglumine ioxitalamate (3.43 ± 1.69; 95% CI: 2.88–3.98), and barium sulfate (5.76 ± 2.08; 95% CI: 5.09–6.43).When compared with the reference standard, the mean number of matching cracks was 4.28 (SD = 1.80) for ioversol, 2.74 (SD = 1.60) for meglumine ioxitalamate, and 4.53 (SD = 1.68) for barium sulfate (Table 1).

**Table 1:** Descriptive statistics for the number of cracks detected per tooth using micro-CT (reference standard) and CBCT under different contrast conditions.

Variable	Minimum	Maximum	Mean	SD
Micro-CT cracks	6.0	40.0	19.92	9.26
Ioversol	2.0	13.0	5.64	2.32
Meglumine ioxitalamate	0	7.0	3.43	1.69
Barium sulfate	1.0	10.0	5.76	2.08
Matching (Ioversol)	1.0	8.0	4.28	1.80
Matching (Meglumine ioxitalamate)	0	6.0	2.74	1.60
Matching (Barium sulfate)	1.0	9.0	4.53	1.68

Micro-CT served as the reference standard for crack detection. Values represent the number of cracks detected per tooth under each imaging condition. “Matching” refers to the number of cracks detected on CBCT that corresponded to cracks identified on micro-CT.

Cracks detected on CBCT that did not correspond to micro-CT findings were considered false-positive detections. The difference between the total number of cracks detected on CBCT and the number of matching cracks represents CBCT findings that were not confirmed by the reference standard.

### Comparison of crack detection among CBCT groups

Since the same specimens were evaluated under each contrast condition, repeated-measures ANOVA demonstrated a significant difference in crack detection among the three contrast agents (*p* = 0.001). Meglumine ioxitalamate detected significantly fewer cracks (mean = 3.43, SD = 1.69) than both ioversol (mean = 5.64, SD = 2.32) and barium sulfate (mean = 5.77, SD = 2.08). Post hoc testing confirmed significant differences between ioversol and Meglumine ioxitalamate (mean difference = 2.20, *p* = 0.001), and between Meglumine ioxitalamate and barium sulfate (mean difference = 2.33, *p* = 0.001). No significant difference was observed between ioversol and barium sulfate (mean difference = − 0.13, *p* = 0.814) (Table 2).

**Table 2:** Comparison of crack detection among CBCT groups.

Group	Mean ± SD	Pairwise comparison	Mean difference	*p*-value
Ioversol	5.64 ± 2.32	vs. Meglumine ioxitalamate	2.20	0.001*
		vs. Barium sulfate	–0.13	0.814
Meglumine ioxitalamate	3.43 ± 1.69	vs. Ioversol	–2.20	0.001*
		vs. Barium sulfate	–2.33	0.001*
Barium sulfate	5.77 ± 2.08	—	—	—

*Repeated measures ANOVA with LSD post hoc test (α = 0.05).

*Significant difference.

### Comparison of matching cracks among CBCT groups relative to the micro-CT reference standard

A statistically significant difference was also found in the number of cracks matching the micro-CT reference among the three groups (*p* = 0.001). Barium sulfate yielded the highest mean number of matching cracks (mean = 4.54, SD = 1.68), followed by ioversol (mean = 4.28, SD = 1.80). Meglumine ioxitalamate detected the fewest matching cracks (mean = 2.74, SD = 1.60). Post hoc testing revealed that meglumine ioxitalamate was significantly less effective than both ioversol (mean difference = 1.53, *p* = 0.001) and barium sulfate (mean difference = 1.79, *p* = 0.001). No significant difference was observed between ioversol and barium sulfate (mean difference = − 0.25, *p* = 0.524) (Table 3).

**Table 3:** Comparison of the number of matching cracks between the three CBCT groups.

Group	Mean ± SD	Pairwise Comparison	Mean Difference	*p*-value
Ioversol	4.28 ± 1.80	vs. Meglumine ioxitalamate	1.53	0.001*
		vs. Barium sulfate	–0.25	0.524
Meglumine ioxitalamate	2.74 ± 1.60	vs. Ioversol	–1.53	0.001*
		vs. Barium sulfate	–1.79	0.001*
Barium sulfate	4.54 ± 1.68	—	—	—

*Repeated measures ANOVA with LSD post hoc test (α = 0.05).

*Significant difference.

### Distribution of crack depth among contrast agents

In a subset analysis of crack depth, barium sulfate revealed the highest proportion of cracks extending beyond the enamel–dentin junction into dentin, representing 20.7% of the total detected cracks. Ioversol accounted for 9.2%, whereas meglumine ioxitalamate detected only 4.6%. These results indicate that barium sulfate may provide superior penetrative and contrast-enhancing properties for identifying deeper cracks.

The visibility of cracks on CBCT images with and without contrast was also assessed. Among all samples, Baseline (unenhanced) CBCT images showed limited crack visualization, with 20.9% of teeth presenting no detectable cracks and only 14% demonstrating visible crack lines prior to contrast application. This comparison highlights the potential diagnostic advantage of contrast agents in improving crack detection.

### Reliability analysis

**Fig. 5:**
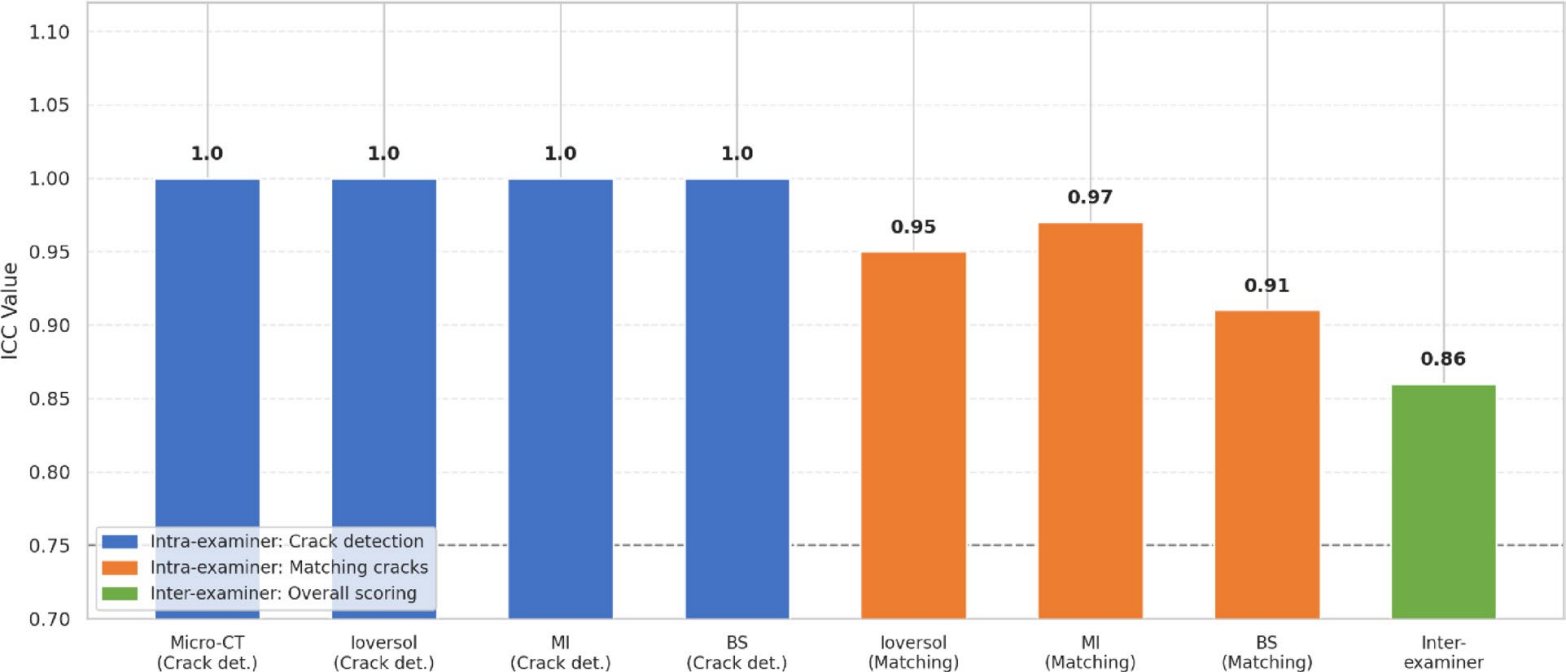
Intra- and inter-examiner reliability for crack detection and crack matching. Bar chart showing intraclass correlation coefficients for intra-examiner test–retest agreement in crack detection across the reference and contrast conditions micro-CT, ioversol, meglumine ioxitalamate, and barium sulfate, and intra-examiner agreement for crack matching after contrast application for ioversol, meglumine ioxitalamate, and barium sulfate, together with inter-examiner agreement for overall scoring. ICCs were calculated using a two-way random-effects model with absolute agreement for single measurements ICC(2,1). Higher ICC values indicate greater agreement.

Intra- and inter-examiner reliability analyses demonstrated excellent consistency (Table 4; Fig. 5). Reliability was assessed using intraclass correlation coefficients (two-way random-effects model, absolute agreement, single measurement; ICC(2,1)). Intra-examiner test–retest reliability for crack detection was perfect across all conditions, with ICC values of 1.00 for micro-CT crack counts and for crack detection following application of ioversol, meglumine ioxitalamate, and barium sulfate. For matching cracks between imaging sessions, intra-examiner ICCs remained excellent for ioversol (0.95), meglumine ioxitalamate (0.97), and barium sulfate (0.91). Inter-examiner agreement was also excellent, with an ICC of 0.86 (*p* = 0.004). These findings indicate a high level of measurement reproducibility between and within examiners.

**Table 4:** Intra- and inter-examiner reliability for crack detection and crack matching.

Outcome	Reliability	Condition	ICC (95% CI)
Crack detection	Intra-examiner	Micro-CT cracks	1.00
Crack detection	Intra-examiner	Ioversol	1.00 (0.09–0.84)
Crack detection	Intra-examiner	Meglumine ioxitalamate	1.00 (0.38–0.92)
Crack detection	Intra-examiner	Barium sulfate	1.00 (0.08–0.85)
Matching cracks	Intra-examiner	Ioversol	0.95
Matching cracks	Intra-examiner	Meglumine ioxitalamate	0.97
Matching cracks	Intra-examiner	Barium sulfate	0.91
Overall scoring	Inter-examiner	All	0.86 (–0.11–0.87)

## Discussion

Diagnosing cracked teeth remains a major clinical challenge because early fractures are subtle and conventional diagnostic methods are limited. CBCT provides high-resolution three dimensional imaging that can reveal crack progression into pulp and root structures, but its ability to detect fine or superficial cracks is limited by resolution thresholds and artifacts that may mimic fractures.^23,29^ In contrast, micro-CT offers superior spatial resolution and was therefore selected as the gold standard in this study. Our protocol employed a 17 μm voxel size, which provides high spatial resolution for detecting dentinal microcracks. Landrigan et al. demonstrated that barium sulfate enhanced micro-CT at ~ 10 μm could visualize subtle cracks not otherwise detectable,^43^ while Wang and Leng confirmed that bone microdamage could be mapped precisely using barium sulfate staining and high-resolution scans. ^44^ Although our voxel size limited detection of the smallest features, micro-CT still revealed substantially more cracks than CBCT with or without contrast agents.

Contrast enhancement improves CBCT crack detection by filling fissures with radiopaque agents, thereby increasing conspicuity. Zhou et al.^23^ demonstrated that ioversol increased sensitivity from 48.4% (unenhanced CBCT) to 67.7% (enhanced). Our results demonstrate a similar diagnostic improvement, with significantly more cracks detected following contrast application. In agreement with Hu et al.,^45^ our study confirmed that Meglumine ioxitalamate improved crack detection compared with conventional CBCT. Overall, these findings indicate that contrast-enhanced CBCT can help overcome important limitations of standard imaging. This observation is supported recently by a systematic review and meta-analysis, which confirmed that contrast-enhanced CBCT significantly increases diagnostic accuracy for detecting cracks and fractures compared with unenhanced imaging.^25^.

Reliability analysis demonstrated high repeatability and reproducibility of the crack scoring method. Intra-examiner ICCs were perfect for micro-CT and for crack detection following application of ioversol, meglumine ioxitalamate, and barium sulfate (ICC = 1.00), while matching cracks between imaging sessions remained highly consistent (ICC = 0.95 for ioversol, 0.97 for meglumine ioxitalamate, and 0.91 for barium sulfate). Group-level intra-examiner ICCs with 95% confidence intervals were 0.624 (0.09–0.84) for ioversol, 0.805 (0.38–0.92) for meglumine ioxitalamate, and 0.644 (0.08–0.85) for barium sulfate. Inter-examiner agreement was also high, with an overall ICC of 0.86 (*p* = 0.004) and a group-level ICC of 0.526 (95% CI − 0.11–0.87, *p* = 0.0036). These results indicate that the scoring criteria were clear and consistently applied, supporting that the differences observed in crack detection among contrast agents reflect true differences in diagnostic performance rather than measurement variability.

Biocompatibility is a key factor in clinical translation. Barium sulfate has consistently demonstrated inert behavior both in vitro and in vivo investigations, supporting its established medical safety^46^. In contrast, iodinated agents such as ioversol and meglumine ioxitalamate have shown dose-dependent cytotoxic effects in vitro,^47,48^ although adverse reactions are uncommon during clinical angiographic use of ioversol^49^. Therefore, while iodinated agents appear to be safe for short diagnostic applications, barium sulfate presently provides the most favorable biological profile. Additional in vivo research is warranted to assess mucosal and pulpal tissue responses following intraoral administration.

Previous investigations have evaluated contrast agents under different experimental conditions—ioversol applied under vacuum,^23^ meglumine diatrizoate by brushing,^13^ and barium sulfate with µ-CT following soaking^45^. The present study is the first to assess all three agents (ioversol, meglumine ioxitalamate, and barium sulfate) using a standardized brushing protocol. Our findings showed that ioversol and barium sulfate achieved comparable performance, whereas meglumine ioxitalamate was less effective. Ioversol, a low-viscosity nonionic agent, penetrates fine fissures effectively. Barium sulfate, although more viscous, adheres well to surfaces and increases radiopacity through particulate deposition^50^. By contrast, the high osmolality of meglumine ioxitalamate may have reduced its penetration by altering fluid dynamics or becoming diluted in artificial saliva^51^.

Overall, this study shows that contrast-enhanced CBCT improves crack detection without compromising image quality or radiation dose, consistent with previous reports. At present, however, no clinical protocols are available for routine patient use. Further work is needed to establish safety, feasibility, and diagnostic accuracy under in vivo conditions before clinical adoption.

### Limitations

This study has several limitations. Although micro-CT was used as the gold standard, the resolution used (17 μm voxel size) may not have allowed visualization of the finest microcracks, and crack width or depth was not quantified, limiting comparisons of agent performance relative to specific crack dimensions. The in vitro design does not replicate the clinical setting, where pulpal pressure, salivary flow, and thermal changes could influence contrast distribution. In addition, outcomes of contrast-enhanced CBCT may vary with scanner model and imaging parameters, and further validation across different systems is required. Biocompatibility was not tested experimentally; while barium sulfate is considered biologically inert and iodinated agents are generally safe at diagnostic doses, their intraoral tolerability requires confirmation in vivo.

In addition, because the contrast agents were applied sequentially to the same specimens, the possibility of order or carry-over effects cannot be completely excluded, although thorough rinsing and overnight storage between cycles were used to minimize residual deposition. Furthermore, the primary outcomes were count-based measures, and although repeated-measures ANOVA was used to compare conditions within the same specimens, alternative statistical models designed for repeated count data may provide a more robust analytical approach in future studies. Despite the use of micro-CT as a reference standard, diagnostic performance metrics such as sensitivity, specificity, and AUROC were not calculated. This limitation arises from the study design, in which all specimens exhibited cracks and analysis was conducted at the crack level, thereby preventing the definition of true negatives and binary outcomes.

In addition, the alignment and matching of CBCT findings with the micro-CT reference standard were performed by a single examiner following consensus evaluation, which may introduce a degree of interpretation bias despite the use of predefined matching criteria.

Future research should address these limitations, establish standardized protocols, and translate laboratory results into clinically applicable methods. It should incorporate both crack-level and tooth-level analyses, including true negative samples, to enable comprehensive evaluation of diagnostic accuracy and improve clinical applicability.

### Clinical implications

Contrast-enhanced CBCT appears to be a valuable supplementary tool for the early detection of cracked teeth, with ioversol and barium sulfate showing the most consistent diagnostic benefit. Once validated under clinical conditions, this technique holds promise for improving endodontic treatment planning and reducing the likelihood of misdiagnosis.

## Conclusion

This in vitro study found that contrast-enhanced CBCT significantly improved the detection of tooth cracks compared with unenhanced imaging. Among the agents tested, ioversol and barium sulfate provided the highest diagnostic performance, whereas meglumine ioxitalamate was less effective, likely due to differences in physicochemical properties such as osmolality and viscosity. Although encouraging, these findings require validation in vivo to account for salivary flow, thermal variation, and functional loading. Future research should also examine the risk of over diagnosis, as well as the practicality, safety, and cost-effectiveness of incorporating contrast-enhanced CBCT into routine endodontic diagnostics.

## Data Availability

The data supporting the findings of this study are available from the corresponding author upon reasonable request.
